# Smoking among older childbearing women - a marker of risky health behaviour a registry-based study in Finland

**DOI:** 10.1186/1471-2458-13-1179

**Published:** 2013-12-13

**Authors:** Reeta Lamminpää, Katri Vehviläinen-Julkunen, Mika Gissler, Seppo Heinonen

**Affiliations:** 1 Department of Nursing Science, University of Eastern Finland, P.O.Box. 1627, 70211 Kuopio, Finland; 2 Department of Nursing Science, University of Eastern Finland and Kuopio University Hospital, Kuopio, Finland; 3 National Institute for Health and Welfare (THL), Helsinki, Finland; 4 Department of Obstetrics and Gynaecology, Kuopio University Hospital and University of Eastern Finland, Kuopio, Finland

**Keywords:** Smoking, Registry-based study, Pregnancy outcome, Older mothers

## Abstract

**Background:**

Smoking during pregnancy is known to negatively affect pregnancy outcomes and it has been associated with numerous complications during pregnancy. Smoking is more common in younger pregnant women, but previous research has shown that adverse pregnancy outcomes related to older maternal age and smoking are even more harmful than with younger smokers. The aim of this study was to compare pregnancy outcomes among smoking and non-smoking pregnant women aged <35 years and ≥35 years.

**Methods:**

In this registry-based study, the data were collected from three national Finnish health registries: Finnish Medical Birth Register, Finnish Hospital Discharge Register, and Register of Congenital Malformations between the years 1997 and 2008. The data included information on 80 260 women who were smoking during pregnancy, of which 11 277 (9%) were ≥35 years and 68 983 (13%) were <35 years old. In multivariate modelling, the main outcome measures were preterm delivery, low Apgar scores at 1 min., low birth weight, small for gestational age, fetal death and preeclampsia.

**Results:**

Fewer older women smoked during pregnancy (9%) than younger women did (13%). Smoking increased the risk of adverse pregnancy outcomes, most in the older group. Multivariate logistic regression using non-smoking women aged <35 years as a reference group indicated that smoking women <35 years had higher rates of preterm delivery (OR 1.27 CI 1.20-1.35), SGA (OR 2.18 CI 2.10-2.26) and LBW (OR 1.73 CI 1.62-1.84).

Non-smoking women ≥35 had higher rates of preterm delivery (OR 1.15 CI 1.10-1.20), fetal death (OR 1.36 CI 1.12-1.64), preeclampsia (OR 1.14 CI 1.09-1.20) and LBW (OR 1.13 CI 1.07-1.19).

Smoking women ≥35 had higher rates of preterm delivery (OR 1.60 CI 1.40-1.82), SGA (OR 2.55 CI 2.34-2.79), fetal death (OR 2.70 CI 1.80-4.05) and LBW (OR 2.50 CI 2.20-2.80).

**Conclusions:**

Smoking during pregnancy increased the risk of adverse pregnancy outcomes in all women, but the rates were the highest for women aged ≥35 years. Pregnant women aged ≥35 years smoking during pregnancy was a distinctly high risk group. Maternity care should identify these women and support them in cessation of smoking during the first trimester of pregnancy.

## Background

Approximately 250 million women worldwide are daily smokers. The prevalence of smoking women is 22% in developed countries and 9% in developing countries. During pregnancy, approximately over 10% of women smoke, and the prevalence of smoking was the highest for women aged 18–19 years old (26%) [1,2].

Smoking during pregnancy is one of the major issues impairing the prognosis of pregnancy [3]. In Finland, the proportion of women who smoke during pregnancy (16%) is still at the same level as in the late 1980s. Smoking during pregnancy is more common in younger women and, in 2012, half of the pregnant women aged under 20 years old smoked during pregnancy. The share was 10% among women aged over 35. In 2012, 42% of all parturients reported quitting smoking during the first trimester of pregnancy, while the number was 16% in 2002 [2].

Smoking during pregnancy has been associated with an increased risk e.g. for miscarriage, ectopic pregnancy, fetal growth restriction, placenta previa, preterm birth, and low birth weight [1,4]. In previous research, prematurity and fetal growth restriction are the areas of most concern. Cessation of smoking during pregnancy results in the reduction of low birth weight, fetal growth restriction, and preterm birth, and thus attributes to a decreased risk for perinatal death and improved neonatal outcomes [1].

It is known that maternal age has increased and continues to grow in many Western countries over the last few years. Several studies have indicated a connection between advanced maternal age of over 35 years and adverse perinatal outcomes as well as an increased risk for certain pregnancy complications. In Finland, the proportion of parturients aged over 35 is increasing and was 19.5% in 2012 [2]. In 2010, the percentages of smoking pregnant women aged over 35 years old were higher for example in Denmark (21%), Luxembourg (24%), Ireland (28%), and Spain (30%), and lower being 11% in Bulgaria and 12% in Poland [5].

Adverse pregnancy outcomes related to older maternal age and smoking have been reported in earlier studies [6-8]. It has been suggested that vulnerability to the negative effects of smoking on birth weight increase with age [8]. Most of the previous studies have concentrated on a few pregnancy outcomes like small for gestational age and low birth weight. In the present study, we compared pregnancy outcomes among smoking and non-smoking pregnant women aged less than 35 years, and 35 or older.

## Methods

The data for this study consist of the information from Finnish Medical Birth Register (MBR), Hospital Discharge Register (HDR), and The Register of Congenital Malformations. Researchers can apply for the authorization for the use of same health register data for scientific research from the register keeping organization (THL National Institute for Health and Welfare), which granted the access to the health register data for this study in September 2009.

The Finnish Medical Birth Register is a population-based registry established in 1987 and is currently compiled by the National Institute for Health and Welfare (THL). The MBR includes information on maternal and neonatal birth characteristics and perinatal outcomes for all women giving birth in Finland and all newborns up to seven days of age. The form is filled by the hospital and sent, mostly electronically, to THL [9].

The Hospital Discharge Register was established in 1969 and it contains information on all aspects of impatient care in public and private hospital visits and outpatient visits to public hospitals (since 1998). Hospitals send data electronically to THL [10].

The Register of Congenital Malformations is run by THL and it contains data on congenital chromosomal and structural anomalies detected in stillborn and live born infants and fetuses in pregnancies terminated due to suspected or confirmed congenital anomaly in Finland nationwide. The Register was established in 1962 and registration of anomaly data began in 1963 [11].

### Participants

**Figure 1:**
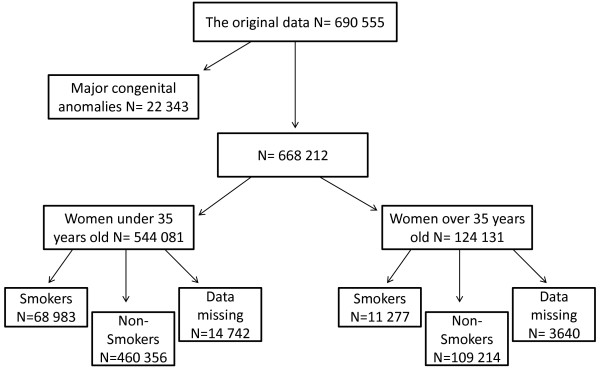
Data flow in the current study.

The original obtained data contains information on 690 555 women and their newborns between the years 1997–2008. The study population of the current study was selected from the original data and included women who were smoking during pregnancy (N = 80 260). Non-smoking women aged less than 35 years old were used as a reference group (N = 460 356). Cases with congenital anomalies were excluded (Figure 1). We compared women aged 35 years or older to women aged less than 35 years old.

Women’s smoking status was classified into four categories: non-smoker, quit smoking after first trimester, smoker, and cases where the smoking status remained unknown. Smoking status was self-reported and only women who were reported to be smokers were included in the smoking category. Women who reported quitting smoking during the first trimester were classified into the non-smoking category.

### Statistical analysis

The statistical analysis was conducted using SPSS for Windows, version 17. The following definitions were used to record pregnancy outcomes: preterm delivery, before 37 weeks of gestation; low Apgar score at 1 min., Apgar score <7; low birth weight (LBW), less than 2500 g; small for gestational age (SGA), infants were considered small when the sex- and age-adjusted birth weight was below the tenth percentile according to the standard tables for the Finnish population data for all births [12].

Statistical differences were evaluated using chi-square tests for dichotomous variables; continuous variables were analyzed using independent sample t-tests. All variables used in the binary logistic regression analysis were dichotomous and missing data for any variable were categorized as “no” (=0). Cases with anomalies were removed from the data. Binary logistic regression adjusted for potential confounding factors included smoking, preeclampsia, IVF, other fertility treatment than IVF, and pre-gravid BMI >25. Because maternal height and weight have been recorded in the data sources only since 2004, logistic regression is presented primarily without BMI. In order to compare the pregnancy outcomes of smoking and non-smoking women aged less than 35 years and 35 or older, we estimated the odds ratios and 95% confidence intervals. Non-smoking women aged less than 35 years was first used as the reference group to compare all four groups [13]. Then smoking women aged 35 years or older were compared separately first to smoking women aged less than 35 years as a reference group and then to non-smoking women aged 35 years or older as a reference group.

## Results

In the original data (N = 690 555), there were 80 260 (12%) women who were smoking during pregnancy, of whom 11 277 (9%) were aged 35 years or older and 68 983 (13%) were less than 35 years old (Figure 1).

The mean ages of the group of women aged less than 35 years old who were smoking was 27.9 (SD 4.7) and with non-smokers 25.7 (SD 3.9), while in the women aged 35 years or older the means were the same for smokers and non-smokers: 37.5 (SD 2.3).

The group of women aged less than 35 years with non-smokers and smokers differed in pre-gravid BMI, late pregnancy bleeding, anemia, fertility treatment other than IVF and IVF, preeclampsia, and being unmarried. The group of women aged 35 years or older with non-smokers and smokers differed in pre-gravid BMI, late pregnancy bleeding, fertility treatment other than IVF and IVF, preeclampsia, and being unmarried.

Table 1 summarizes the background information of the four groups.

**Table 1:** Background information of non-smoking and smoking pregnant women aged <35 years and ≥35 years old

	**<35y non-smoker**	**Smoker**	***P***	**≥35y non-smoker**	**Smoker**	***P***
Maternal diabetes (N/%)	15834 (3.4)	2471 (3.6)	<.056	6535 (6.0)	715 (6.3)	<.129
Pregravid BMI >25 kg/m^2^ (N/%)	49107 (29.4)	8049 (35.0)	<.001	14483 (37.1)	1529 (45.2)	<.001
Placenta previa (N/%)	935 (0.2)	158 (0.2)	<.162	457 (0.4)	44 (0.4)	<.657
Late pregnancy bleeding (N/%)	6578 (1.4)	1141 (1.7)	<.001	1826 (1.7)	250 (2.2)	<.001
Anemia (N/%)	3327 (0.7)	579 (0.8)	<.001	855 (0.8)	68 (0.6)	<.037
Unmarried (N/%)	156358 (34.0)	43740(63.4)	<.001	30367(27.8)	5829 (51.7)	<.001
Fertility treatment other than IVF (N/%)	6233 (1.4)	348 (0.5)	<.001	3264 (3.0)	139 (1.2)	<.001
IVF (N/%)	5753 (1.2)	297 (0.4)	<.001	3553 (3.3)	141 (1.3)	<.001
Preeclampsia (N/%)	21954(4.8)	2400(3.5)	<.001	6400(5.9)	535(4.7)	<.001

The mean weight before pregnancy in the group of under 35-year-olds was 66.4 kg (SD 15.1 kg) in smokers and 65.8 kg in non-smokers (SD 13.3 kg). The mean weight in the group of women aged 35 years or older was 70.0 kg in smokers (SD 15.1 kg) and 68.4 kg in non-smokers (SD 13.6 kg). The mean birth weight was 3532 g (SD 568 g) in women aged less than 35 who were non-smokers and 3358 g (SD 573 g) in smokers. In the group of smoking women aged 35 years or older, the mean birth weight was 3302 g (SD 651 g) and 3536 g (SD 618 g) in non-smokers.

**Figure 2:**
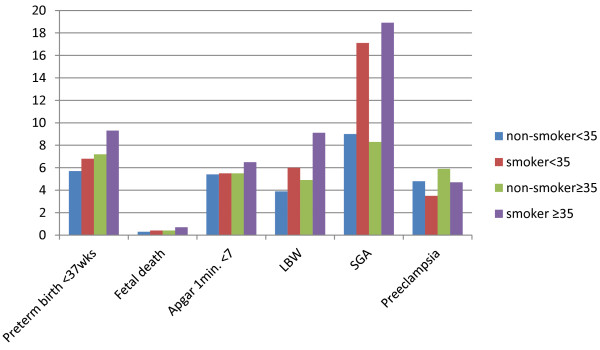
Percentages of outcomes of non-smoking and smoking women aged <35 years and ≥35 years old.

Percentages of outcomes among non-smoking and smoking women aged less than 35 years and 35 years or older are shown in Figure 2.

The incidence of preterm delivery, SGA and LBW was higher in pregnant women aged less than 35 years old who smoked. Non-smoking women aged 35 years or older had higher rates of preterm delivery, fetal death, preeclampsia and LBW compared to non-smoking women aged less than 35 years (Table 2).

**Table 2:** Pregnancy outcomes in non-smoking and smoking women aged <35 and ≥35 years old

**Outcome**	**Adjusted OR and 95% CI**			
	**<35non-smoker**	**<35smoker**	**≥35non-smoker**	**≥35smoker**
Preterm delivery <28wk	1	1.29, 1.27-1.34 (*1.27, *1.20-1.35)	1.17, 1.14-1.21 (*1.15, *1.10-1.20)	1.73, 1.61-1.85 (*1.60, *1.40-1.82)
SGA(<90th percentile)	1	2.14, 2.09-2.19 (*2.18, *2.10-2.26)	0.91, 0.89-0.93 (*0.91, *0.88-0.95)	2.38, 2.27-2.51 (*2.55, *2.34-2.79)
Low birth weight (<2500 g)	1	1.74, 1.68-1.80 (*1.73, *1.62-1.84)	1.15, 1.11-1.19 (*1.13, *1.07-1.19)	2.60, 2.43-2.78 (*2.50, *2.20-2.80)
Fetal death	1	1.39, 1.22-1.58 (*1.18, *0.92-1.15)	1.37, 1.23-1.53 (*1.36, *1.12-1.64)	2.57, 2.06-3.22 (*2.70, *1.80-4.05)
Low Apgar score (<7) at 1 min.	1	1.03, 1.00-1.07 (*1.02, *0.96-1.01)	0.97, 0.94-1.00 (*0.95, *0.90-0.99)	1.21, 1.11-1.30 (*1.12, *0.97-1.30)
Preeclampsia	1	0.73, 0.70-0.76 (*0.78, *0.72-0.84)	1.20, 1.17-1.24 (*1.14, *1.09-1.20)	1.00, 0.91-1.09 (*0.90,*0.77-1.06)

(*BMI included).

In the group of smoking women aged 35 years or older, SGA, fetal death and LBW were found to occur twice as often compared to non-smoking women aged less than 35 years old. The incidence of preterm delivery was significantly higher as well (Table 2).

Comparison between smoking women aged 35 years or older and smoking women less than 35 years is shown in Table 3, where the impact of maternal age was also found to be significant among smoking women with regard to preterm delivery, SGA and low birth weight. In the same vein of thought, the differences between women aged 35 years or older compared and their non-smoking counterparts preterm delivery, low Apgar scores at 1 min., LBW, SGA and fetal death were all found to be higher in the smoking women (Table 4).

**Table 3:** Outcomes of <35 year-old smoking and ≥35 year-old smoking women

**Outcome**	**<35 smokers**	**≥35 smokers**	**Adjusted OR and 95% CI**
Preterm delivery (before 37 wk)	4425 (5.9%)	975(1.3%)	1.35, 1.25-1.45 (*1.29, *1.12-1.48)
SGA (<90^th^ percentile)	11430 (14.7%)	2043 (2.6%)	1.12, 1.07-1.19 (*1.18, *1.07-1.29)
Low birth weight (<2500 g)	4120(5.1%)	1027 (1.3%)	1.51, 1.41-1.62 (*1.50, *1.31-1.71)
Fetal death	273 (0.3%)	83 (0.1%)	1.87, 1.46-2.40 (*2.36, *1.49-3.73)
Low Apgar score (<7) at 1 min.	3589 (4.7%)	692 (0.9%)	1.17, 1.08-1.27 (*1.09, *0.94-1.27)
Preeclampsia	2400 (3.0%)	535 (0.7%)	1.37, 1.24-1.50 (*1.17, *0.98-1.39)

(* BMI included).

**Table 4:** Outcomes of non-smoking and smoking women aged ≥35 years old

**Outcome**	**≥35 non-smokers**	**≥35 smokers**	**Adjusted OR and 95% CI**
Preterm delivery (before 37 wk)	7399 (7.2%)	975 (9.3%)	1.44, 1.34-1.55 (*1.34,*1.17-1.53)
SGA (<90^th^ percentile)	8693 (8.3%)	2043(18.9%)	2.65, 2.51-2.80(*2.83, *2.58-3.12)
Low birth weight (<2500 g)	5337 (4.9%)	1027 (9.1%)	2.20, 2.06-2.37(*2.15, *1.88-2.46)
Fetal death	438 (0.4%)	83 (0.7%)	1.85, 1.46-2.34(*1.93, *1.26-2.96)
Low Apgar score (<7) at 1 min.	5667 (5.5%)	692 (6.5%)	1.24, 1.14-1.34(*1.18, *1.02-1.36)
Preeclampsia	6400 (5.9%)	535 (4.7%)	0.83, 0.66-0.93 (*0.78, *0.76-0.91)

(* BMI included).

## Discussion

The aim of this study was to examine pregnancy outcomes among smoking and non-smoking pregnant women aged less than 35 years and 35 years or older.

There were fewer women who were smoking during pregnancy among women aged 35 years or older that in the group of women under 35, but the former groups’ behavioural risk profile and outcome results clearly demonstrated that older smoking women are a distinct high risk group.

Smoking increased the risks of preterm birth, SGA, LBW, and fetal death in all pregnant women, but even more so in older women.

Maternal age of 35 years or more independently of smoking appeared to increase the risks of preterm delivery, fetal death, preeclampsia and LBW. However, the combination of maternal age of 35 years or more and smoking was significantly increasing the risks of these outcomes in the subgroup analyses performed, showing that the two independent risks, smoking and advanced maternal age, are additive. This reflected two important health issues. First, advanced maternal age alone increased the risk of adverse pregnancy outcome. Second, the high risk group identified by maternal smoking was smaller but even less health conscious in the case of older than younger women. In other words, smoking is a more powerful marker of risky health behaviour among older than younger women.

The incremental risk caused by smoking was clearly higher in older than in younger women in all studied outcomes, even though the effect on fetal growth was clinically the most important.

Our findings are in line with previous studies reporting adverse pregnancy outcomes in smoking women [4,6-8,14] and especially with the one reporting that the smoking-related risk of SGA increases with maternal age [7,14]. Smoking during pregnancy is an important health problem associated with adverse outcomes, particularly fetal growth restriction and preterm birth, which can both have far-reaching health consequences into adult life [15].

The toxic effects of smoking may influence fetal growth more among older smoking women and the longer exposure in the mother to the harmful effects of smoking may also be more damaging to the fetus [7,14].

In the present study, older women who were smoking were more often overweight than their non-smoking counterparts, but they had had less infertility treatments than non-smokers. On the other hand, smoking and obesity are known to impair fertility. The discrepancy between the known increasing rate of fertility problems and the observed underuse of fertility treatments probably reflected the fact that smokers were less health conscious and sought health care less often than their non-smoking counterparts. In the present study, the study population of the older smoking pregnant women seems to have been positively selected due to their successful pregnancy in spite of the smoking-related risks affecting fertility altogether.

In the present study, smokers were more often unmarried. Married adults are considered to be in better health than unmarried ones and the economic situation of unmarried women is also likely to be worse than of those who are married. Poor health consciousness and smoking are related to being less well-educated and more often unemployed with more alcohol consumption, pregnancy terminations, and untreated infections [16,17].

It has been reported that women who smoke during pregnancy tend to under-report their smoking [18]. Maternal under-reporting has significant implications on the validity of research in this area, producing underestimates of smoking prevalence and overestimates of the non-smoking/quit smoking status [19]. It is also possible that older pregnant women report smoking more accurately than younger ones [8]. However, the strength of this study is a large population-based register data comprising almost 700 000 births. It has been shown in several studies comparing the internal validity that, in Finnish health registries, the validity and coverage are good as all events are included in the data and the registries comply with reality [20]. The information of health registries provide a highly complete and high-quality source of information that can be utilized, for example, in scientific research [21].

It has been suggested that smoking acts as a marker for other unhealthy habits, exposures, or differences in behaviour or socioeconomic status, but they cannot be measured using birth certificates or registries [8]. However, in Finland, pregnant women are relatively homogenous and they receive similar antenatal and obstetric care, in which case the influence of socioeconomic factors on pregnancy outcome is limited.

The results of the current study suggest and confirm earlier implications that, especially from a public health viewpoint, more attention should be paid to older smoking women during their pregnancy due to an increased risk for impaired fetal growth and preterm delivery as well as other adverse pregnancy outcomes that are harmful to the newborn. In one study, 70% of those who smoked during their first pregnancy continued smoking in a sequential pregnancy, which highlights the importance of smoking cessation during the first pregnancy [22].

## Conclusions

Cessation of smoking should be monitored and support should be given during the first trimester of pregnancy especially to the older smokers. It can be presumed that older smoking pregnant women smoke while consciously ignoring the pregnancy-related risks. Therefore, the cessation of smoking post pregnancy is unlikely. Maternity care should identify and concentrate on these groups of women who clearly have health behavioural issues that can harm pregnancy.

## Competing interests

The authors declare that they have no competing interest.

## Authors’ contributions

RL conceived the study with contributions from SH and KVJ. RL prepared the data and performed the statistical analysis. RL and SH interpreted the results with contributions from KVJ and MG. RL reviewed the literature and wrote the manuscript. SH, KVJ and MG critically revised the manuscript for scientific quality and content. All authors approved the final version for publication.

## Pre-publication history

The pre-publication history for this paper can be accessed here:

http://www.biomedcentral.com/1471-2458/13/1179/prepub
